# Artificial Intelligence in Colonoscopy: A Tool, Not a Necessity for Experts

**DOI:** 10.7759/cureus.93046

**Published:** 2025-09-23

**Authors:** Raveena Karthikeyan, Kristin Delfino, Bridget Mcclain, Sowmyanarayanan Thuppal, V Prasad Poola

**Affiliations:** 1 Surgery, Southern Illinois University (SIU) School of Medicine, Springfield, USA; 2 Center for Clinical Research, Southern Illinois University (SIU) School of Medicine, Springfield, USA; 3 Surgery, University of Kentucky College of Medicine, Lexington, USA; 4 Colorectal Surgery, Southern Illinois University (SIU) School of Medicine, Springfield, USA

**Keywords:** adenoma detection rate, artificial intelligence, colonoscopy, colon polyp, experienced endoscopist

## Abstract

Introduction: Artificial intelligence (AI) tools for polyp detection during colonoscopy have shown promise in improving polyp detection rate (PDR) and adenoma detection rate (ADR). However, in real-world settings, the results are inconsistent, particularly among experienced endoscopists. This study evaluates the impact of AI on ADR and PDR when used by a single high-volume, experienced endoscopist.

Methods: A retrospective analysis was conducted from July 2019 to June 2023, involving colonoscopies performed by a single high-volume endoscopist. Patients undergoing screening or surveillance colonoscopy were included, while those with diagnostic indications were excluded. Outcomes were compared between two groups: pre-AI-assisted colonoscopy (pre-AIAC) (July 2019-June 2022) and AI-assisted colonoscopy (AIAC) (July 2022-June 2023).

Results: Of 1,273 colonoscopies, 814 met the inclusion criteria. Quality metrics, including the Boston Bowel Prep Score and withdrawal time greater than six minutes, were comparable between the groups. ADR was 151 (35%) in the pre-AIAC group and 116 (31%) in AIAC group, with no statistical significance (p = 0.2). Similarly, PDR, 184 (42%) vs. 145 (38%); p = 0.22, and adenomas per colonoscopy, 0.62 (1.23) vs. 0.51 (0.97); p = 0.22, showed no statistically significant differences. However, the mean withdrawal time was significantly shorter in the AIAC group (9.34 vs. 10.44 minutes; p < 0.0001).

Conclusion: AIAC may not significantly improve adenoma or PDRs when performed by an experienced endoscopist. However, it was associated with a significant reduction in mean withdrawal time. Further research is needed to explore these findings in broader clinical settings.

## Introduction

Colorectal cancer is the second leading cause of cancer-related deaths in the USA [1]. Over the past few decades, the incidence of colon and rectal cancers has declined, primarily due to the widespread adoption of screening colonoscopy [2]. For colonoscopy to be an effective preventive measure, a thorough, high-quality examination of the colonic mucosa is essential to detect and remove polyps. Advancements in colonoscopy techniques and the implementation of quality metrics have significantly improved mucosal visualization, polyp detection, and removal.

A recent innovation in this field is the integration of artificial intelligence (AI) to enhance polyp detection and reduce the likelihood of missed lesions. Among the key quality indicators of colonoscopy, adenoma detection rate (ADR) remains paramount, as higher ADRs have been associated with a reduced risk of interval colorectal cancer and mortality [3,4]. Recent studies have demonstrated AI's ability to improve ADR during colonoscopy [5-8]. However, other studies suggest that its benefits may be limited, particularly among experienced endoscopists [9-11].

Despite these mixed findings, real-world data on AI-assisted colonoscopy (AIAC) continue to emerge, while its adoption in clinical practice accelerates. This study aims to assess the impact of AI on ADR and polyp detection rate (PDR) when used by an experienced, high-volume endoscopist. Unlike previous studies involving multiple endoscopists, our approach minimizes interoperator variability and potential bias, providing a clearer evaluation of AI's effectiveness in one high-volume expert endoscopist.

## Materials and methods

Following institutional review board approval, we conducted a retrospective study using prospectively collected data from July 2019 to June 2023. The study involved a single fellowship-trained colorectal surgeon with nine years of experience performing 400-500 colonoscopies annually. Notably, a real-time AIAC device (GI Genius™ Intelligent Endoscopy Module by Medtronic, Minneapolis, MN) was introduced in the endoscopy suite in July 2022. All subsequent colonoscopies utilized AI, creating a natural cohort of AI-assisted colonoscopies (AIACs).

The study included patients undergoing colonoscopy for colorectal cancer screening, including those with a positive fecal immunochemical test and those undergoing colon polyp surveillance. Exclusion criteria included colonoscopies performed for diagnostic purposes (symptomatic patients, known inflammatory bowel disease, IBD, or established malignancy), incomplete colonoscopies, and surveillance colonoscopies following colon resection.

The patients were categorized into two groups: Pre-AIAC group (colonoscopies performed from July 2019 to June 2022 without AI assistance) and AIAC group (colonoscopies performed from July 2022 to June 2023 with AI assistance). Data collected included baseline patient characteristics (age, gender, American Society of Anesthesiologists, ASA, grade), polyp characteristics (location, size, pathology), and withdrawal time for both groups. The primary outcome was to compare the ADR between the two groups. Secondary outcomes included comparisons of PDR and adenomas per colonoscopy (APC).

Statistical analyses were conducted between the Pre-AIAC and AIAC groups across patient characteristics, procedural quality parameters, and outcomes. Continuous variables analyzed included age (years), withdrawal time (minutes), number of polyps, and number of adenomas. Categorical variables analyzed included gender (men vs. women), ASA grade (1, 2, 3, 4), indication (screening vs. surveillance), Boston Bowel Prep Score (BBPS <6 vs. ≥6), and polyp and adenoma detection (yes vs. no), polyp size (<5, 5-10, >10 mm), polyp location (right colon, transverse, left colon, rectum), and removal method (biopsy forceps vs. snare).

Each colonoscopy was treated as a single record for detection rate analysis, while each detected polyp was analyzed individually for polyp characteristics. Given the retrospective nature of the study, no formal sample size calculation was performed. Instead, we utilized all available cases meeting the inclusion criteria. Each study group included over 350 cases, providing a robust dataset for analysis.

Continuous variables are reported with measures of central tendency (mean, median) and dispersion (range, standard deviation, SD). Differences between groups were analyzed using the t-test or Mann-Whitney test, depending on data distribution. Categorical variables are presented as frequencies and percentages, with comparisons made using Pearson's chi-square test or Fisher’s exact test as appropriate. A p value of <0.05 was considered statistically significant. Statistical analyses were conducted using Statistical Analysis System version 9.4 (SAS Inc., Cary, NC).

## Results

**Figure 1:**
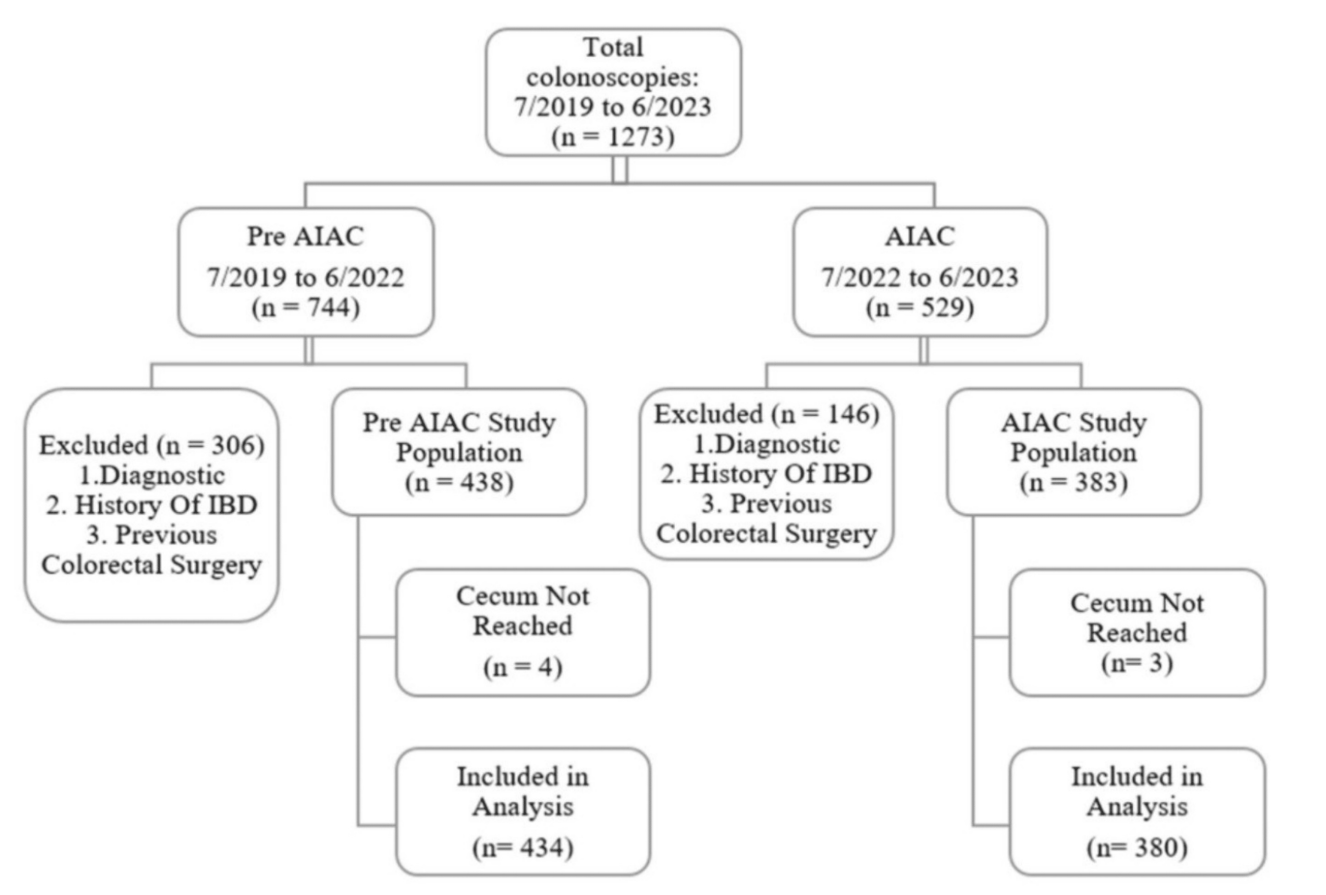
Schematic of study population

Out of 1,273 colonoscopies performed during the study period, 821 met the initial inclusion criteria after excluding 452 cases (35.5%) conducted for diagnostic purposes, known inflammatory bowel disease (IBD), or prior colorectal surgery. Additionally, seven incomplete colonoscopies were excluded, resulting in a total of 814 colonoscopies for final analysis: 434 colonoscopies in the pre-AIAC group and 380 in the AIAC group (Figure 1).

Both groups were comparable in age: mean (SD): 57 (8.7) vs. 56 (7.8); p = 0.13; gender distribution: 240 (55%) vs. 202 (53%) men; p = 0.54; and ASA grades (p = 0.21) (Table 1). However, there was a significant difference in the proportion of patients undergoing colonoscopy for screening vs. surveillance, 336/98 (77%/23%) in pre-AIAC vs. 248/132 (65%/35%) in AIAC; p = 0.0001. Other quality metrics, including the BBPS and withdrawal time greater than six minutes, were comparable between the groups (Table 1). However, the mean withdrawal time was significantly longer in the pre-AIAC group (10.44 vs. 9.34 minutes; p < 0.0001).

**Table 1:** Patient characteristics and quality metrics ^*^Four colonoscopies with missing ASA grades ^**^25 colonoscopies with missing BBPS ^***^Six colonoscopies with missing withdrawal time AIAC: artificial intelligence-assisted colonoscopy; SD: standard deviation; ASA: American Society of Anesthesiologists; BBPS: Boston Bowel Prep Score

Patient characteristics and quality metrics	Pre-AIAC (n = 434)	AIAC (n = 380)	p value	Test statistic	Test
Age, mean (SD)	57 (8.7)	56 (7.8)	0.13	-1.5	Mann-Whitney
Gender, male, n (%)	240 (55%)	202 (53%)	0.54	0.37	Chi-square
ASA grade^*^, n (%)					
1	26 (6%)	33 (9%)	0.21	4.58	Fisher's exact test
2	290 (67%)	265 (70%)			
3	111 (26%)	81 (21%)			
4	3 (1%)	1 (<1%)			
Indication, n (%)					
Screening	336 (77%)	248 (65%)	0.0001	14.77	Chi-square
Surveillance	98 (23%)	132 (35%)			
Quality parameters					
Boston bowel prep score^**^					
Poor (1-5), n (%)	11 (3%)	7 (2%)	0.44	0.58	Chi-square
Good and very good (6-9), n (%)	401 (97%)	370 (98%)			
Withdrawal time >6 minutes, n (%)	425 (98%)	371 (97%)	0.82	0.05	Chi-square
Mean withdrawal time in minutes^***^ (SD)	10.44 (5.32)	9.34 (4.16)	<0.0001	-4.29	Mann-Whitney

Of the 814 colonoscopies analyzed, 329 (40%) resulted in polyp detection, with a total of 608 polyps identified. There was no statistically significant difference in the distribution of polyp sizes or location between the pre-AIAC and AIAC groups, as listed in Table 2. Notably, there was a statistically significant difference in the method of polyp removal between the pre-AIAC and AIAC groups (p < 0.0001). In the AIAC group, a greater proportion of polyps were removed using biopsy forceps, 149 (61%) vs. 140 (40%), whereas snare was more common in the pre-AIAC group, 214 (60%) vs. 95 (39%).

**Table 2:** Characteristics of polyps ^*^10 colonoscopies with a missing method of removal AIAC: artificial intelligence-assisted colonoscopy

Polyp characteristics	Pre-AIAC (n = 360 polyps)	AIAC (n = 248 polyps)	p value	Test statistic	Test
Size of polyp, n (% of polyps)					
<5 mm	99 (28%)	72 (29%)	0.24	2.89	Chi-square
5-10 mm	231 (64%)	146 (59%)			
>10 mm	30 (8%)	30 (12%)			
Location of polyps, n (% of polyps)					
Right colon	96 (27%)	73 (29%)	0.7	1.43	Chi-square
Transverse	94 (26%)	70 (28%)			
Left colon	127 (35%)	79 (32%)			
Rectum	43 (12%)	26 (11%)			
Method of removal of polyps, n (% of polyps)^*^					
Biopsy forceps	140 (40%)	149 (61%)	<0.0001	26.78	Chi-square
Snare	214 (60%)	95 (39%)			

The adenoma detection rate (ADR) was 151 (35%) in the pre-AIAC group and 116 (31%) in the AIAC group, though the difference was not statistically significant (p = 0.2). Similarly, secondary outcomes, including PDR, 184 (42%) vs. 145 (38%); p = 0.22, and APC, 0.62 (1.23) vs. 0.51 (0.97); p = 0.22, showed no statistically significant differences between the groups (Table 3).

**Table 3:** Polyp detection between the pre-AIAC and AIAC groups ^*^Two colonoscopies with missing pathology AIAC: artificial intelligence-assisted colonoscopy; SD: standard deviation; PDR: polyp detection rate; ADR: adenoma detection rate; APC: adenomas per colonoscopy

Polyp detection between pre-AIAC and AIAC group	Pre-AIAC (n = 434)	AIAC (n = 380)	p value	Test statistic	Test
Total number of polyps, n	360	248	-	-	-
Total number of patients with polyps, n (%) PDR	184 (42%)	145 (38%)	0.22	1.51	Chi-square
Mean number of polyps per colonoscopy (SD)	0.83 (1.43)	0.65 (1.06)	0.17	-1.37	Mann-Whitney U
Total number of adenomas^*^, n (% of polyps)	269 (75%)	195 (79%)	0.25	1.32	Chi-square
Total number of patients with adenomas^*^, n (%) ADR	151 (35%)	116 (31%)	0.2	1.67	Chi-square
Mean number of adenomas per colonoscopy APC^*^ (SD)	0.62 (1.23)	0.51 (0.97)	0.22	-1.21	Mann-Whitney U

## Discussion

The findings of our study indicate that the use of AI provided no significant benefit in improving ADR or PDR when performed by an experienced endoscopist. However, it shortened the withdrawal time without compromising the quality of colonoscopy.

Prior studies on screening colonoscopy have shown that up to 25% of colorectal neoplasms are missed by endoscopists, contributing to an increased incidence of interval colorectal cancer, estimated at 0.5-1 per 1,000 person-years [3]. A key factor in these missed neoplasms is the failure to recognize polyps during colonoscopy. The integration of AI has been proposed as a tool to enhance the efficacy of colonoscopy by mitigating errors related to operator fatigue and other human biases [3]. AI-assisted polyp detection systems, developed using deep neural networks that mimic the complex structure of the human brain, have been shown to have theoretical and practical advantages. Hence, several AI-assisted detection systems such as GI-Genius, EndoBRAIN, CAD-EYE, EndoScreener, EndoAngel, Endo-AID, CADDIE, WISE VISION, and ME-APDS are available for both research and commercial purposes. Among these, GI-Genius is the most widely available and commonly used in the United States [12].

Recent studies have increasingly explored the impact of AIAC on ADRs; however, findings from real-world practice remain mixed. A 2021 meta-analysis of 10 randomized control trials (RCTs) by Huang et al. reported AI's ability to enhance both adenoma and PDRs [5]. Similarly, Xu et al.'s multicenter RCT reported that AIAC increased ADR to 39.9%, compared to 32.4% with conventional colonoscopy (p < 0.001), with benefits observed between both expert and nonexpert endoscopists [6]. Koh et al.'s prospective cohort study further supported these findings, showing ADR improvement even among experienced operators [7]. Makar et al. also reported that AIAC significantly removed adenoma detection but not sessile serrated detection in all endoscopists irrespective of their experience [8]. Repici et al.'s studies have further highlighted AI's advantages in detecting smaller and distal adenomas [13,14]. In contrast, other studies have raised concerns about the generalizability of these findings, particularly among experienced endoscopists. Levy et al.'s retrospective study found a lower ADR in the AI-assisted group compared to conventional colonoscopy, along with shorter overall procedure times [9]. Similarly, Nehme et al. reported no improvement in ADR with AIAC in daily clinical practice among endoscopists with high baseline ADRs [10]. Wei et al.'s RCT also reported that AIAC did not result in a statistically significant difference in the ADR but noted an increased identification of nonadenomatous nonserrated polyps per colonoscopy [11]. Given these discrepancies, the present study was conducted using a single experienced endoscopist to minimize interoperator variability and potential sources of bias.

Our study found no significant differences in ADR, PDR, APC, or polyp size and location between the pre-AIAC and AIAC groups. These findings support the notion that AI is a tool without significant benefits for experienced endoscopists. A common concern among endoscopists is that AIAC may prolong withdrawal time due to a higher rate of false positives, requiring additional time for lesion verification [6,11]. However, our study, in contrast, demonstrated a shorter mean withdrawal time in the AI-assisted group. This finding may be explained by the following factors: first, the conventional colonoscopy group in our study had a significantly higher rate of snare polypectomies, which likely extended withdrawal time; second, AI may assist in promptly identifying polyps that were initially detected but inadvertently overlooked during deployment of forceps or snare for polypectomy, potentially saving time; third, the pre-AIAC and AIAC groups were evaluated across different time periods, which raised the possibility of improvement in endoscopist’s experience over time rather than the use of AI assistance.

To our knowledge, this is the only study comparing colonoscopy quality with and without AI assistance, focusing on a single endoscopist to minimize variability and bias. Nevertheless, the study has several limitations. First, this is a retrospective, nonrandomized design, which limits our ability to establish a causal relationship. Although there were no statistically significant differences in quality parameters such as the BBPS or withdrawal times greater than six minutes, there was a significant difference in the proportion of patients undergoing screening vs. surveillance colonoscopy between the two groups, which could introduce selection bias. Additionally, improvements in endoscopists’ experience over time could potentially contribute to the shorter withdrawal time observed in the AIAC group. To mitigate these biases, future randomized controlled studies involving multiple experienced endoscopists should be considered. Furthermore, the study was conducted by a single experienced endoscopist, which enhances procedural consistency but may limit generalizability to less experienced endoscopists or trainees. Another limitation is the lack of blinding, which could have influenced the endoscopist's behavior. However, this reflects real-world clinical practice, where blinding is often not feasible.

## Conclusions

Our study concludes that AIAC may not significantly improve adenoma or PDRs when performed by an experienced, high-volume endoscopist in clinical practice. However, this finding is not applicable to novice endoscopists or fellows in-training and is beyond the scope of the paper. Notably, our study also finds that AIAC was associated with a reduction in withdrawal time without comprising the quality, even among experienced endoscopists. This time-saving benefit has the potential to enhance workflow efficacy in busy endoscopy units. To further validate this finding and assess their broader applicability in the real-word settings, multi-institutional prospective study involving a diverse group of experienced endoscopists is warranted.
